# Congenital unilateral facial palsy revealing a facial nerve agenesis: a case report and review of the literature

**DOI:** 10.1259/bjrcr.20180029

**Published:** 2018-07-13

**Authors:** Yaotse Elikplim Nordjoe, Ouidad Azdad, Mohamed Lahkim, Laila Jroundi, Fatima Zahrae Laamrani

**Affiliations:** ^1^Imaging Department, Ibn Sina Hospital, Rabat, Morocco

## Abstract

Facial nerve aplasia is an extremely rare condition that is usually syndromic, namely, in Moebius syndrome. The occurrence of isolated agenesis of facial nerve is even rarer, with only few cases reported in the literature. We report a case of congenital facial paralysis due to facial nerve aplasia diagnosed on MRI, while no noticeable abnormality was detected on the temporal bone CT.

## Differential diagnosis

Congenital facial paralysis (CFP) is uncommon and may cause multiple problems for the newborn, such as difficulty with nursing and incomplete eye closure. It is classified as traumatic or developmental; unilateral or bilateral; and complete or incomplete.^1^

It is important to differentiate between birth trauma-related and developmental causes of CFP, because it serves as a cornerstone in planning the treatment and informing the parents about the prognosis. History and physical examination can guide the diagnosis. However, in some cases; and despite all efforts, MRI is required.

## Clinical presentation

A 5-years old girl, born at full term by normal vaginal delivery not involving forceps, was presented with a left side facial paralysis. There was no history of birth trauma or facial paralysis in her family. She had facial paralysis affecting all sectors of the left facial nerve. Physical examination showed a facial asymmetry, incomplete left eye closing, right deviation of the angle of mouth, and left-sided loss of nasolabial furrow. No impairment of other cranial nerves is found, namely, there is no gait or mobility disorders on both side (Cochlea-vestibular nerves are normal).

## Imaging findings

Temporal bone CT revealed no noticeable abnormality.

**Figure 1.**
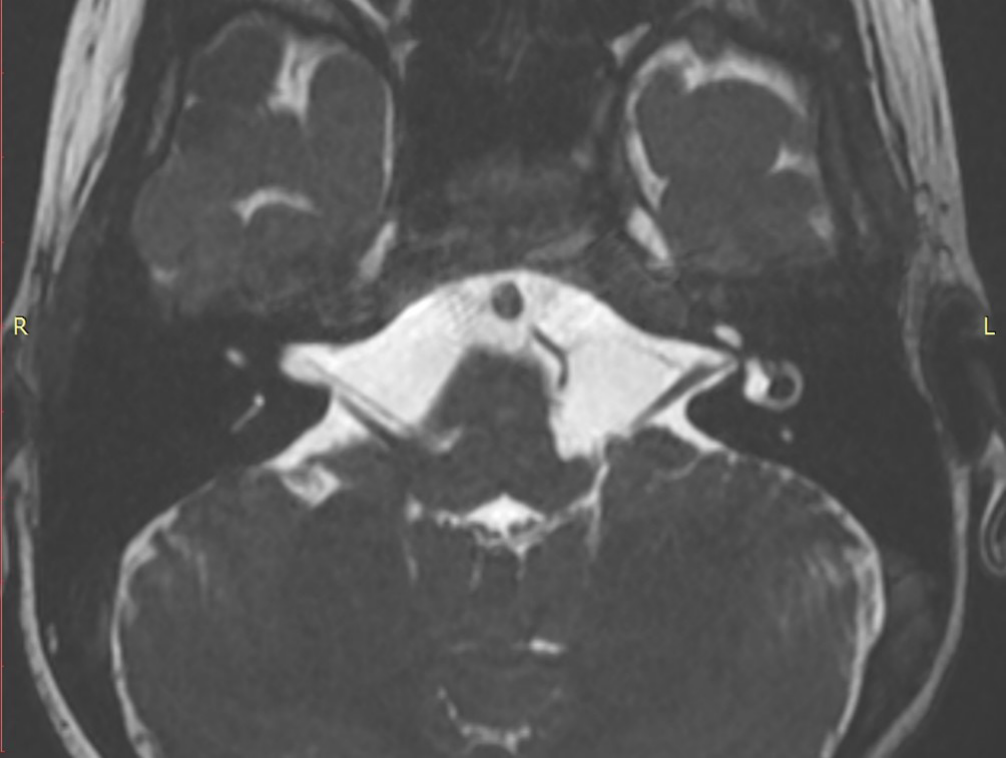
High resolution 3D *T*_2_W sequence (3D-FIESTA), axial reconstruction, shows an absence of left facial nerve. 3D, three-dimensional; *T*_2_W, *T*_2_ weighted.

**Figure 2.**
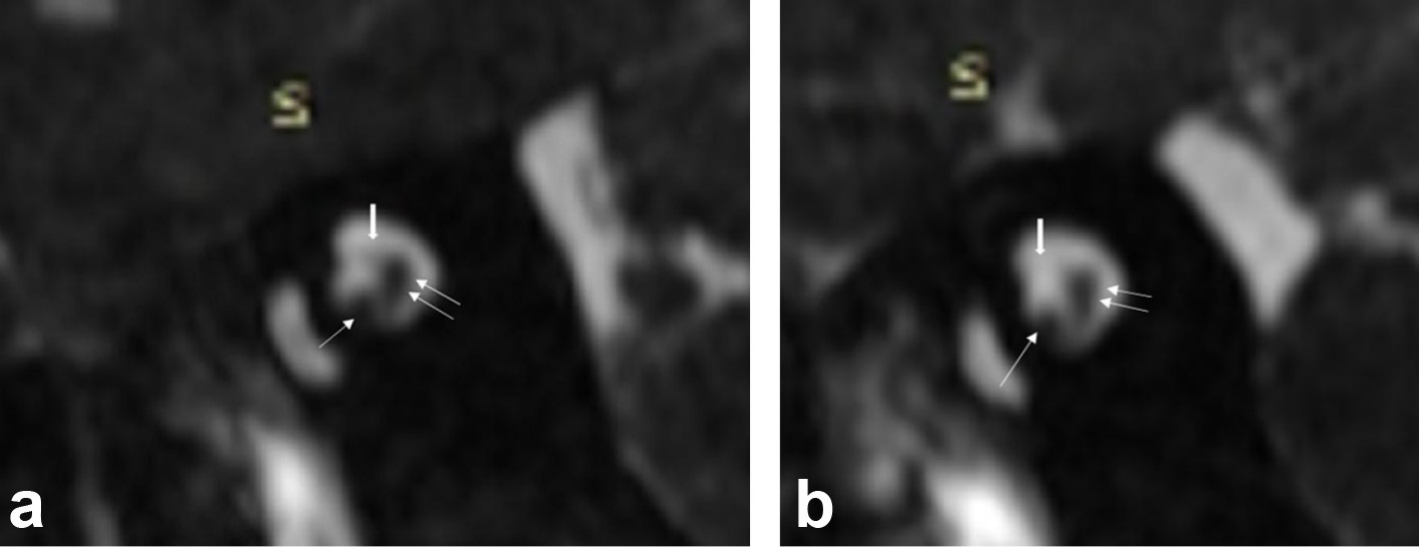
(a) Right side: high resolution 3D *T*_2_W sequence (3D-FIESTA), Sagittal-Oblique reconstruction perpendicular to the right internal auditory canal. The vestibular nerves (superior and inferior) in the posterior aspect of the internal auditory canal, appear as a whole in a “comma shape-like image” and is indeed two nerves together (double arrow). The facial nerve lies in the anterosuperior aspect of the internal auditory canal (bold arrow). The Cochlear nerve is located in the anteroinferior aspect of the internal auditory canal (single arrow). Normal 4/4 aspect. (b) Left side: high resolution 3D *T*_2_W sequence (3D-FIESTA), Sagittal-Oblique reconstruction perpendicular to the left internal auditory canal. The anterior-superior aspect of the internal auditory canal is empty (bold arrow), corresponding to a complete absence of the facial nerve. Cochlear (single arrow) and vestibular nerves (double arrow) are normal. Abnormal 3/4 aspect due to the facial nerve agenesis. 3D, three-dimensional; *T*_2_W, *T*_2_ weighted.

An MRI was performed using 1.5 T superconducting system (GE Healthcare, Optima MR360). Routine MR sequences were performed as per institutional protocol, which include fast spin echo *T*_1_ (sagittal), *T*_2_ (axial, coronal,), fluid-attenuated inversion recovery (coronal) and diffusion-weighted imaging. In addition, a high resolution *T*_2_ W stack of images [three-dimensional (3D)-FIESTA] was also obtained to evaluate facial nerve and other cranial nerves. The 3D-FIESTA sequences (Figures 1, 2a,b), showed the absence of the left facial nerve throughout its course, while no abnormality was found on the right side. The rest of the cranial nerves were normal. Parotid glands were normally seen. On the basis of the clinical and MRI features, the diagnosis of isolated congenital facial nerve agenesis was then retained.

## Discussion

CFP is an uncommon situation in new borns with an occurrence of 2 per 1000 live births. The major role of the physician is to differentiate between traumatic and developmental aetiologies. This distinction is important not only for the treatment options and prognosis but may have medicolegal implications as well.^2^

Developmental aetiologies include very rarely, isolated cases of aplasia/hypoplasia of facial nerve or their nuclei, or more commonly, various syndromic associations such as Poland syndrome (congenital facial palsy with absent pectoralis major muscle), Moebius syndrome (variable degree of agenesis/hypoplasia of the sixth and seventh cranial nerves/nuclei), Goldenhaar's syndrome (unilateral facial hypoplasia), and cardiofacial syndrome (weakness of the facial muscles).^3^

There are very few cases of isolated facial nerve agenesis/hypoplasia described in the literature. Facial nerve hypoplasia has also been reported to be associated with abnormalities of other cranial nerves (especially vestibulocochlear),^4^ and ipsilateral parotid gland agenesis.^5^ The first case of isolated complete congenital facial nerve agenesis was reported by Jervis et al in 2001, where diagnosis was made incidentally during a surgical procedure for an unrelated condition, on a 7-year-old child with a congenital facial palsy, diagnosed at birth, who subsequently developed a non-tuberculous mycobacterial infection of the ipsilateral parotid gland.^6^ Recently, in 2016, Kumar et al^7^ published a two case-report article of non-syndromic facial nerve agenesis in two infant, depicted on MRI which, was described back then as a novelty.

A normal temporal bone CT-scan cannot rule out a facial nerve abnormality—as seen in the present case. Brainstem hypoplasia, abnormal facial nucleus, agenesis of the facial nerve may be seen in MRI with a normal or abnormal CT. Internal auditory canal aplasia or stenosis may also be diagnosed in CT or MRI.^8^

MRI is the reference diagnostic modality to evaluate the cisternal and canalicular facial nerve segments. It is best evaluated on high-resolution *T*_2_ weighted 3D sequences, *e.g.* 3D-FIESTA (GE Healthcare), 3D-CISS (Siemens) or B-FFE (Philips). These sequences combine heavily *T*_2_ weighted images with high spatial resolution, allowing thin-slice images (0.3–0.8 mm) that enable reconstructions if necessary, allowing a detailed exploration of the cranial nerves, throughout their course.^9^ A cross section sagittal oblique slice, perpendicular to the nerves axis allows a good evaluation of the nerve’s intracanalicular portion. A thinning or an absence of facial nerve within this canal matches respectively the diagnosis of hypoplasia or aplasia. Associated aplasia/hypoplasia of other cranial nerves, especially vestibulocochlear nerve, should also be looked out for.^10^

The high-resolution *T*_2_ weighted 3D MRI sequence (3D-FIESTA) confirmed the agenesis of the left facial nerve in our case. Analysis of the other cranial nerves especially the trigeminal (V), abducens (VI), right facial (VII), and acoustic nerve (VIII) showed no abnormalities.

Most patient presenting a congenital unilateral facial nerve palsy due to a birth trauma usually recover within a few months. Facial nerve agenesis along with other developmental causes of congenital facial palsy usually ensue poor prognosis, and a spontaneous recovery cannot be expected.^11^ However, a mild but noticeable improvement of facial functions or residual activity in some facial muscles, were described in patients with facial nerve agenesis. This phenomenon is explained by the presence of aberrant innervations of some of the facial muscles by other cranial nerves such as the trigeminal, hypoglossal, or glossopharyngeal nerves.^9^

## Learning points

1. Congenital unilateral facial nerve palsy without birth injury is rare, and usually of unknown aetiology.
2. It can be isolated or associated to other malformative syndromes such as Moebius syndrome, Poland syndrome or Goldenhaar syndrome.
3. The distinction between a congenital and traumatic facial palsy is important not only for treatment options and prognosis but may have medicolegal implications as well.
4. High-resolution *T*_2_ weighted 3D MRI sequence (3D-FIESTA, 3D-CISS, B-FFE) can enable the radiologists and neurologists to procure this rare diagnosis which helps in clinical management and prognosis.
